# A Practical Treatise on the Diseases of Children

**Published:** 1846-10-01

**Authors:** 


### A Practical Treatise on the Diseases of Children. By *James Milman Coley*, M.D. 8vo. pp. 460. London, 1846.

Dr. Coley states that he has been induced to publish his book for the purpose of presenting "to the Medical Profession and the Public a comprehensive work on the Diseases of Infants and Children, which the physician, the surgeon, and the general practitioner may consult as a work of reference." We are much pleased in being able to state that the words "the Public" in the above sentence form a mere superfluity, introduced we suppose to round a period, for the misgivings we had that they were intended to imply an attempt at the baneful practice of popularizing medical science, now so much in vogue, have not been confirmed by a perusal of the work, which is in fact solely addressed, as it ought to be, to the medical profession itself. We wish we were enabled also to state that the anticipations of its becoming a work of reference, entertained by its author, were likely to be realised. To constitute it such a much greater degree of completeness than at present characterizes it would be necessary. We do not mean this as regards the number of subjects treated of, the whole of these being apparently noticed: but the symptoms, diagnosis, and pathology of many, and indeed of most, of them are far too superficially dwelt upon to allow of the work superseding those now in use. Not to mention foreign ones, we consider Evanson and Maunsel's Treatise a very superior production to the present. Dr. Coley states that Treatises on Diseases of Children are defective in not embodying accounts of maladies of a surgical nature, and he attributes this to most of the works in question having been written by physicians. He, having been engaged in general practice in the country prior to taking his degree (as indeed have many of our most esteemed physicians), and seen much surgery in a populous district, has "enjoyed singular opportunities of observing the origin and progress of surgical as well as medical cases, and acquiring that discrimination and manual dexterity, which are necessary qualifications in any one who undertakes to instruct others on subjects requiring a practical knowledge of both branches of the profession." We were not aware before, that "manual dexterity" was one of the necessary qualifications of a writer, even upon surgery, and certainly not for the description of the very limited amount of operative surgery which the diseases of children call for. However, since the author placed such stress upon this characteristic of his treatise, we turned to those portions of it which especially relate to surgery, and can only say that, if preceding writers upon Diseases of Children have not bestowed equal attention upon this branch of the subject, they might have done so with very little trouble to themselves, inasmuch as these consist chiefly of quotations from the works of well-known writers, as *e. g.* Little upon Club Foot, Lawrence, Middlemore and Walker on Diseases of the Eyes, Liston and Velpeau upon the operation for Strabismus, &c. &c.

But not only do we think Dr. Coley's work is a superficial one, and in no-wise entitled to rank as one of reference, seeing that it brings forward no large amount of original facts and observations, or contains anything like a complete summary of those already known; but we find it defective in another point of view, namely, in neglecting to specify the relative frequency of the occurrence of the diseases treated of in children, as compared with adults, and in the not sufficiently distinguishing the modifications of treatment some of these should receive in these young subjects. In fact, the whole catalogue of ills that flesh is heir to is studiously gone through, but the special adaptation of much of the information adduced to the complaints of children is not easily perceived. In proof of what we say, we will take an example or two at random. Thus, treating of *Mercurial Salivation,* the author, never remarking upon its rarity in children, gives us a description of the symptoms as they are observed in adults, and, among these, adverts to the fatal erethismus described by Pearson. He then tells us that salivation is best relieved by eight or ten leeches applied beneath the maxilla, followed by a soft poultice. This is quoted from Dr. Watson, but suitable as it may be for adults it would often prove death to a child. Then we are recommended to use a lead gargle—that is, we suppose, when we can get the child to employ it. In *Pneumonia,* too, we have all the symptoms of the disease as it occurs in adults (among these as an essential one, the rusty expectoration, as if expectoration of any kind were common in a child) given as a matter of course, but none of those especially observed in the child. In regard to the pathology of the disease the author is more successful in establishing the requisite distinctions, and then only because he furnishes long extracts from Rilliet and Barthez's masterly work: but when he speaks of treatment he certainly describes such only as is fitted for adults. Thus we are told the child is to be freely bled from the arm or jugular vein in the sitting posture to syncope, and that, if the treatment has been delayed, blood will have to be abstracted several times (!) to secure relief. Calomel and antimony are afterwards to be put into requisition, suspending the former, however, "when the gums are seriously affected." It is true, the author observes, that some young children, as well as adults, will not bear this treatment, and many require leeching only; but he evidently believes these are mere exceptions. Indeed, he cautions us not to be deceived by apparent debility consequent on pulmonary and cerebral congestion, found in some cases, in which the heart's action becomes almost suspended, and the patient apparently dying. This condition is to be managed by first giving four or five grains of sesquicarbonate of ammonia until some regularity of circulation is produced, and then bleeding; but the detection of the patient's true state in his apparent deliquium requires a greater nicety of touch than many we fear will possess, when the pulse of a child is in question. By its careful examination the attendant will perceive "that the calibre of the artery and the firmness or density of its muscular coat are undiminished, and that the obscure and undulating impulse affords really the perception of a struggle in the heart to carry on the circulation, rather than any deficiency in the quantity of blood in the circulation." Those who know how difficult it is decide in some of these cases in the adult, can best appreciate its frequent impossibility in the child. However, none can doubt the propriety of the temporary exhibition of stimuli, whether in apparent or real deliquium. Throughout his work the author far too indiscriminately recommends free bleeding, seeming to be unaware of the conditions of children which contra-indicate this. We some time since ridiculed the *hematophobia* displayed by Mr. Hood, in his recent book on the diseases of Children; but when we find such rash procedures countenanced in a work purporting to be one of reference on the subject, we must allow that his is the least injurious of the two extremes.

Other examples of these imperfections might be cited, but we prefer placing before our readers some of the remarks which Dr. Coley considers as of an original character; and, indeed, while we deny that his work possesses the complete and practically useful character which he seems to claim for it, we are free to confess it contains several interesting observations, evidently the production of a reflecting mind. He regards as a mere vulgar prejudice the influence which is so generally attributed to the process of *Dentition* in disordering various portions of the economy. "It is lamentable to notice the ignorance displayed by the profession as well as the public on this subject; every concomitant disease, the exact nature of which is not obvious to their apprehension, being attributed to the teeth." Treating of diarrhœa he thus speaks regarding the effect of teething.

"I may, however, observe that purging, which happens to concur with dentition, has no necessary connection with that process. I have already explained, under the head of 'Dentition,' the effect produced upon the alimentary canal by the growth and production of the primary teeth, which is the very opposite to that of excitement. When dentition happens to be proceeding with any remarkable activity, particularly in delicate children, the processes of digestion, chylification, and even the peristaltic action of the bowels are interrupted in the same ratio, and the whole chylopoietic system rendered torpid. Hence, instead of purging, we shall always find a state of constipation prevailing, together with inaction of the liver, until the deciduous teeth are unfolded, and the delicate animo-chemical process of depositing the enamel, which requires so much organic influence, has been completed. When, therefore, mucous, muco-purulent, or purulent diarrhœa occurs during dentition, it may always be traced to chronic inflammation in the mucous follicles of the villi, produced by cold, as will be explained in the next chapter. Another striking proof that diarrhœa, and other inflammatory diseases in the bowels of infants and children, under two years of age, do not proceed from the excitement of dentition, is the fact, that whenever such diseases do occur, the process of dentition is interrupted as long as such diseases continue; as may be observed by the defective construction of the primary teeth, which happen to be forming at the time, and particularly the deposit of enamel, which, after remittent fever, severe diarrhœa, or marasmus, will be found as soon as the teeth have completely emerged from the gums by the subsequent growth of the fangs, disfigured with defects in the enamel, consisting of its total absence in transverse patches corresponding in extent with the duration and severity of the contemporaneous intestinal disease. Notwithstanding these obvious facts, writers on the diseases of children, both British and Foreign, concur in labouring to prove the correctness of their mistaken views and inverted pathology, by contending that the mucous follicles during infancy undergo rapid development in the intestines, and that they supply the sudden and immense secretion of serous fluid occurring in diarrhœa, and thus act as a salutary check to the excitement of dentition. These pathologists, in their desire to blame the teeth for every disease appearing during the earlier periods of life, quite forget that inflammatory diarrhœa and dysentery attack individuals at all ages, even those who have shed their secondary as well as their primary teeth; and that in all the same disorganizations are discovered after death as those, which are met with in children, who happen to die before primary dentition is completed." P. 200.

With the opinions here expressed, and repeated on various occasions throughout the work, we cannot agree. The coincidence of profuse diarrhœa with difficult dentition, and of its arrest upon the teeth coming through, is of too frequent occurrence to allow of our denying their relation of cause and effect. That the diarrhœa too is not an inflammatory affection seems to us proved by its rapid induction and cessation, its frequent alternation with the non-inflammatory secretion of the salivary glands, and the measures which most effectually relieve it. Of the dependence upon, or at all events the aggravation of many, and indeed most, of the chronic, and some of the acute diseases of childhood by difficult dentition, and of the great utility of freely lancing the gums in such cases we entertain no doubt whatever, and can only wonder at any being raised by an experienced practitioner.

*Erysipelas of Infants.*—Dr. Coley observes that, not only his own observation, but the descriptions of writers upon the diseases of children, prove that this disease, which usually attacks the abdomen, thighs or nates of infants, is identical in its nature with the phlegmonous erysipelas of adults. By the action of cold the vessels of the cellular adipose membrane have become obstructed, and suppuration and gangrene, if relief be not obtained, are the consequences. This disease has no analogy with superficial erratic erysipelas, or with the blush upon the abdomen which is sometimes symptomatic of erythematous inflammation of the intestinal mucous membrane.

"*Treatment.*—Instead of trifling with the application of starch powder, or flour, as recommended by some writers on this subject, until disorganization of the cellular membrane, or gangrene, has taken place, we should make free incisions through the nodules, and indurated crimson-coloured parts, deep into the cellular and adipose membrane, which decided practice will instantly arrest the local disease, and prevent the typhoid fever and destruction of parts, which would inevitably follow. This practice is applicable in every situation, in which the disease may appear; and will always prevent that dreadful destruction of parts, which authors describe as resulting from the disease. The amazing enlargement of the scrotum, produced by the induration and infiltration of the loose cellular membrane of this part, added to its dark purple colour, are calculated to alarm and deter an inexperienced practitioner from adopting the practice I have recommended, and found invariably successful. The incisions, however, must be deep and unsparing; otherwise the patient will be lost by impending gangrene, and its accompanying typhoid fever. Every hard crimson nodule, in particular, should be freely divided, as that is otherwise destined to certain destruction, and contains the elements of spreading mischief; as I have already explained, in unfolding the pathology of the disease in the article, 'Phlegmonous Erysipelas.' The only applications required after the incisions have been made, will be evaporating poultices, and afterwards folds of linen rag, moistened with warm water. The physician must not place confidence in the antiseptic properties of quina, or any other medicine, but should immediately avail himself of the surgical assistance which alone can save the patient. When proper local treatment is adopted in due time, little medicine will be required; but the vessels should not be permitted to remain unrelieved by the knife, until their contents have been effused and converted into pus; for this timid and dilatory practice will only assist nature in completing her work of destruction." P. 375.

The author makes no mention of the danger of and means of arresting hæmorrhage consequent upon these free incisions. We have seen more than one *adult* sink under its influence.

*Furunculus or Boil.*—"I consider this disease nothing more than an aggravated and severe form of erythema nodosum. It consists of a phlegmonous inflammation in the cellular and adipose membrane, in which the same phenomena occur, on a more limited scale, as in phlegmonous erysipelas, to which the reader is referred. The obstruction in the small vessels circulating in the cellular membrane, occasioned by the application of cold, produces the death of that structure, as far as the disease extends, and the blood extravasated from the over-distended and ruptured capillaries becomes coagulated, and undergoes a conversion into pus-globules. The mode by which nature expels the dead mass is by external progressive absorption, on the same principle that she contrives an outlet for any other extraneous matter.

"*Treatment.*—Liquor potassæ, sarsaparilla, and various other remedies, have been mentioned by writers and lecturers for the treatment of this troublesome disease; but all who speak of it confess its obstinacy and the inefficiency of remedies. The only medicine which exerts a specific action on the disease is *bichloride* of *mercury*, which should be given in small doses. For instance, one-eighteenth of a grain in a mixture twice a day, to a child four or five years old, and one-twelfth in a mixture or pill, to a child from six to twelve years. This medicine will not only shorten the progress of the disease, when suppuration is inevitable, but will, when used sufficiently early, prevent that termination; and put a stop to the disposition to generate successive crops of boils, which, as I have before stated, are often found to torment the patient during many months. When evident derangement in the stomach and bowels exists, a dose of chloride of mercury and jalap may be given with advantage every third morning. The best local treatment, in the first instance, will be the frequent application of warm water, and when suppuration has commenced, a common poultice, which should be continued until the slough has exfoliated; after which nothing more will be required than a fold of linen, moistened with warm water, until cicatrization takes place." P. 119.

*Treatment of Porrigo Scutulata, or Ringworm of the Scalp.*—"The astonishing number of specifics recommended for this eruption, prove the intractable nature of the disease. Very few of the acrid applications mentioned in books are advisable or useful. The hair should be cut off, not shaved; and during the day time folds of linen rag, moistened in cold water, should be applied all over the head, and moistened again with cold water as often as they become dry. At bed time the head should be covered with the leaves of ivy (hedera helix.) The Irish or giant ivy, from the Canaries, is the best. The patient should take internally a grain or two of iodide of potash, twice a day, and be well purged with salts and senna every second or third morning. I have never found this treatment to fail, except in one case, which was afterwards cured by an ointment composed of one drachm of sulphate of iron, and one ounce of lard; the cold water being used at the same time. The manner in which the water dressing acts, is by promoting evaporation, which removes the excessively redundant heat from the surface; and I suppose the ivy-leaves, possessing a kind of natural varnish, operate by exciting cutaneous perspiration, which of itself is a cooling process. This is the only mode of treatment from which I have found success in a reasonable time." P. 88.

*Dysentery in Children.*—When this is in the acute inflammatory form leeches to the abdomen and a saline aperient are indicated. The sulphate of magnesia forms the best aperient. It may be given in doses of from ten grains for an infant to half-a-drachm for a child of three or four years, and repeated every four hours until the pain and purging subside. Opium and astringents are here hurtful; but tenesmus may be relieved by warm water enemata or immersion in the hip-bath. A few drops of tincture of gentian may in this case too be added to the saline aperient. The *congestive* form of the disease, in which the vital powers are eminently collapsed, is one of great danger. The heat of surface and the circulation must be restored by blankets, hot flannels, warm baths, and repeated small doses of calomel and opium. A generous diet and stimuli must also be resorted to, and, if the tenesmus is severe, an opiate injection should be given. As soon as re-action is restored, a few grains of rhubarb and magnesia may be given every few hours; and when the heat of skin becomes anormally high, and there is discharge of blood and pain, small local depletions may be resorted to. For *chronic* remittent and intermittent dysentery, the small doses of sulphate of magnesia given three times a day suffice to effect a cure.

*Treatment of Pertussis.*—Dr. Coley protests against the use of the numerous poisonous and powerful remedies recommended in the treatment of this disease, and believes it may, as a general rule, be cured by much milder measures. A *temperature* of 65°, maintained day and night, is that upon which he chiefly relies. It should be the same in the bed-room and sitting-room, these being on the same floor when possible. The respective rooms should be well ventilated during the patient's absence from either, but the windows of the room he is in must not be opened. The bowels are to be regulated, which, with a mixture of citrate of potash and squill, will constitute all the medical appliances during the first stage. During the second stage, the cough will be found much milder than it would have been had the child been exposed to the air; and in from six to eight weeks all symptoms of the disease will have disappeared. This regulation of the temperature may be commenced at any stage of the disease, while the cough is alarming, and the expectoration copious, nor will it interfere with any treatment the special exigencies of the case may demand. If the patient is already suffering from hectic and purulent expectoration, the regulated temperature and half or a grain of sulphate of zinc with half-a-grain of quinine dissolved in water, will relieve him speedily. Concurrent phlegmasiæ must be treated by depletion, but they will never occur when this temperature has been adopted from the beginning.

The importance of a regulated temperature in this disease is, we believe, generally acknowledged; but the difficulty, or indeed the impossibility, of maintaining it in the houses of the great mass of patients is too obvious to need comment; and in these cases it is a fortunate thing when the progress of the disease can be curtailed, as it often can, by prussic acid or some other of the substances stigmatized by the author.

*Plugging the Nares in Epistaxis.*—"Pressure may be effectually applied by means of a soft bougie, a ligature, and a small piece of sponge. It should first be ascertained whether the bleeding proceeds from one or both of the nostrils. When both nostrils bleed, two pieces of sponge will be required. The patient being seated with his head held backwards, the surgeon should fasten one end of the ligature to the bougie near its smaller extremity. The bougie must then be introduced along the floor of the nostril in a horizontal direction, carrying one end of the ligature with it, till its point is visible in the fauces behind the soft palate. The bougie being gently pushed towards the back of the fauces, the ligature may be seized by a pair of common forceps and brought out through the mouth, when it should be cut away from the bougie, which should be withdrawn. The end of the ligature which has been brought through the mouth must now be formed into a noose, into which the sponge should be fixed. Lastly, the other end of the ligature left hanging out at the nostril must be drawn tight, so as to bring the sponge in close contact with the posterior opening, and tied firmly to a roll of linen placed in front of the nostril. If the sponge is well adapted to the size of the posterior aperture in the fauces, the bleeding will immediately cease after the ligature is fastened. The same process must be adopted at the other nostril when the hemorrhage arises from both. The sponge should be removed on the third or fourth day by quietly pushing it into view by means of a bougie, and taking hold of it with the forceps; care being taken to prevent its slipping into the pharynx by retaining the ligature in the other hand during its return through the mouth. This operation is much more easy of execution both to the patient and surgeon than the common and ineffectual practice of stuffing the nostrils with lint." P. 244.

From the specimens we have exhibited, and which might be extended had we more space, our readers will perceive that the work, however defective as a whole, contains some interesting matter.

